# Using weight loss to predict outcome and define a humane endpoint in preclinical sepsis studies

**DOI:** 10.1038/s41598-024-72039-1

**Published:** 2024-09-10

**Authors:** Maëlick Brochut, Tytti Heinonen, Tiia Snäkä, Charly Gilbert, Didier Le Roy, Thierry Roger

**Affiliations:** Infectious Diseases Service, Department of Medicine, Lausanne University Hospital and University of Lausanne, CLED.04.407, Chemin des Boveresses 155, 1066 Epalinges, Switzerland

**Keywords:** Sepsis, Infectious disease, Weight loss, Preclinical, Humane endpoint, Immunology, Infection, Infectious diseases

## Abstract

Preclinical mouse models are critical for understanding the pathophysiological response to infections and developing treatment strategies for sepsis. In keeping with ethical values, researchers follow guidelines to minimize the suffering of the mice. Weight loss is a criteria used as a humane end point, but there is no official recommendation for a maximum weight loss leading to euthanasia. To evaluate whether the thresholds used in daily practice are optimal, we performed a comprehensive retrospective analysis of data generated over 10 years with > 2300 mice used in models of infection with *Listeria monocytogenes*, *Streptococcus pneumoniae*, *Candida albicans* and H1N1 influenza virus. Weight loss segregated mice that survived from those that did not. Statistical analyses revealed that lowering the weight loss thresholds used (none, 30% or 20%) would have increased mortality rates due to the sacrifice of mice that survived infections (*p* < 0.01–0.001). Power calculations showed high variability and reduction of power as weight loss thresholds approached 20% for *S. pneumoniae* and *L. monocytogenes* models. Hence, weight loss thresholds need to be adapted to each model of infection used in a laboratory. Overall, weight loss is a valuable predictor of mortality that contributes to the robustness of composite scores. To our knowledge, this is the most extensive study exploring the relationship between weight loss threshold and sepsis outcome. It underscores the importance of the infection-model-specific evaluation of weight loss for use in clinical scores defining humane endpoints to minimize mouse suffering without compromising statistical power and scientific objectives.

## Introduction

Animal experimentation is a complementary pilar to in silico, in vitro and ex vivo analyses in advancing our understanding of the regulation of biological processes and the development of diseases. Rodents, mainly mice, account for around 95% of the animals that are utilized in biomedical research^1^. The use of mice has become widespread due to: (1) the relatively ease of obtaining large numbers, (2) the panel of congenic and genetically modified strains, (3) genetic similarities to humans, (4) the wealth of knowledge gained over decades of research, and (5) adaptability to controlled experimental conditions. Research using rodents has been instrumental to decipher host pathogen interactions and to advance therapeutics for human health such as antimicrobials and immunomodulatory therapies^2^.

A conscientious approach to preclinical research involves a commitment to the principles of the 3Rs (replacement, reduction, refinement). 3Rs advocate the ethical treatment of research animals and aim to minimize their suffering^3^. Scoring systems based on subjective and objective parameters such as behavior, external clinical signs, body weight and body temperature have been developed to scale rodent welfare and include a humane endpoint, i.e. a cut-off value at which animals are euthanized to avoid excessive suffering^4,5^. Scoring systems are established in collaboration with local veterinary and ethics authorities, adapted to the research theme (oncology, behavior, etc.) and updated to reflect changes in local legislation and ethical guidelines^6^. Body weight loss is one of the main criteria to evaluate the well-being of mice, but its usage either alone or in combination with other criteria is debated^7–12^. There is no official weight loss recommendation for euthanasia, but a threshold of 20% is often used^13^.

The innate immune system provides the first line of defense against infections, and a dysregulated host response underlies severe infections and sepsis^14^. Sepsis, defined as a life-threatening organ dysfunction caused by an infection^15,16^, is responsible for one in five deaths worldwide. Recently, the world health organization recognized sepsis as a global priority and antimicrobial resistance (a contributor to sepsis) as one of the top ten threats to global health. Following these initiatives, Switzerland launched the Swiss sepsis national action plan, identifying four priorities among which research development^17^. Participating to that effort, our laboratory has a long-standing expertise in studying host–pathogen interactions and testing biomarkers and innovative therapies for infected patients^18–20^. As an example, our work on the cytokine macrophage migration inhibitory factor (MIF) led to the identification of small molecules inhibitors of MIF^21,22^, and the development of humanized anti-MIF monoclonal antibodies that have been evaluated in phase 1/2a clinical trials^23^.

In daily practice, we use models of infections induced by bacteria, viruses and fungi inoculated through different routes to induce peritonitis, pneumonia and systemic infection. We adapt the inoculum to induce either a moderate or a severe infection, and we use genetic and pharmacological approaches to modulate host defenses. To take all these conditions into account, a weight loss of 20% or 30% was recently adopted as a humane endpoint in agreement with regulatory authorities. To assess whether these thresholds were optimal, we performed comprehensive retrospective analyses of data obtained over 10 years and with more than 2300 mice used in models of infections with *Listeria monocytogenes*, *Streptococcus pneumoniae*, *Candida albicans* and H1N1 influenza virus (H1N1). We observed that reducing the weight loss thresholds leading to euthanasia substantially increased mortality and decreased statistical stability and power in a model-dependent manner. Our data suggest that weight loss thresholds need to be adapted to each infectious model so that the composite clinical scores including weight loss minimize mouse suffering without jeopardizing statistical power and scientific objectives.

## Methods

### Mouse models of infection

**Fig. 1:**
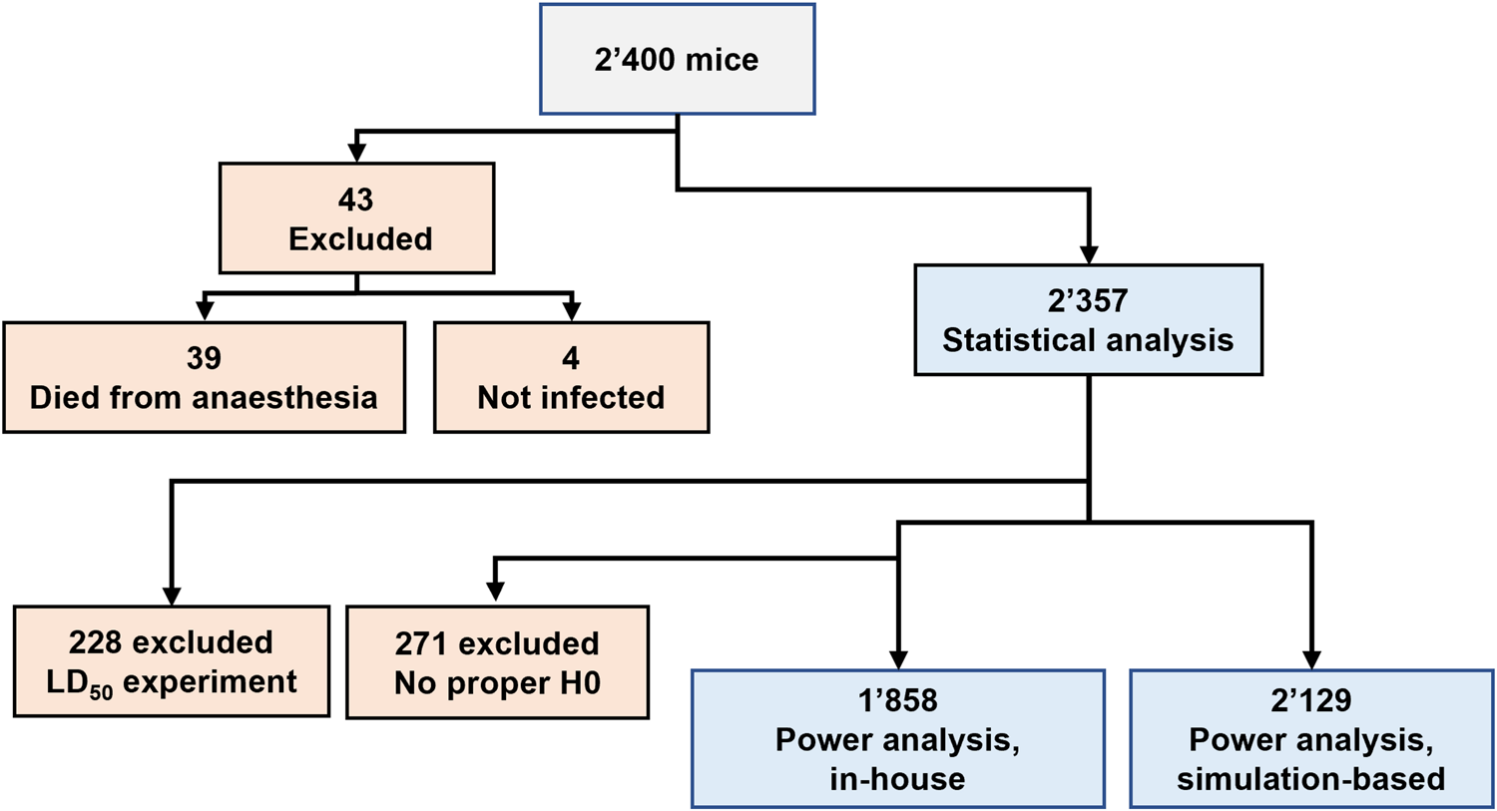
Number of mice used in statistical and power analyses. Experiments were conducted from June 2014 to March 2023. Exclusion criteria included death just following anaesthesia (n = 39) and incorrect injection of the infectious agent (n = 4). Statistical analyses were performed on 2'357 mice. Power analyses were performed on 1858 and 2199 mice for the in-house and simulation-based calculations. Experiments without an appropriate ${H}_{0}$ (n = 271) or determining LD_50_ (n = 228) were excluded from in-house power analysis.

No mice were used for this study. Data were retrieved from experiments performed in 2014–2023 and approved by the Veterinary Affairs Department of the *Direction Générale de l’Agriculture, de la Viticulture et des Affaires Vétérinaires* of Etat de Vaud (Saint-Sulpice, Switzerland) (Refs.^24–36^ and unpublished data). Experiments were performed with *Streptococcus pneumoniae* American type collection (ATCC) 6303 and H1N1 PR08 (Porto Rico 08) IV inoculated intranasally under anesthesia with ketamine/xylazine, and *Listeria monocytogenes* 10403S and *Candida albicans* ATCC 90028 inoculated intravenously. Among 2400 used mice, 43 were excluded from our analyses (Fig. 1). Experiments were designed to determine lethal dose 50 (LD_50_) (n = 228 mice) or to compare at least two experimental conditions (n = 2129 mice). Altogether, 2024 C57BL/6J mice and 333 BALB/c mice were used (Fig. 1).

### Score sheets

In vivo experiments involved regular recording of the mice using preestablished score sheets. The experiments performed with authorizations covering 2014 to 2018 (cohort 1) had no limit on weight loss (Supplementary Table 1). Visual criteria were used to assess the well-being of the animals, with scores ranging from 0 (normal) to 4 (moribund). A score of 4 led to mouse euthanasia. The mice were observed at least once daily regardless the severity score. The weight was measured at the same time as the score was evaluated. The experiments performed with authorizations covering 2018 to 2023 (cohort 2) combined objective and subjective criteria (Supplementary Table 2). The combined score led to different actions: monitor mice 3 times a week, once a day or twice a day, and euthanasia. On its own, a weight loss ≥ 20% led to the euthanasia of mice infected with *L. monocytogenes*, and a weight loss ≥ 30% led to the euthanasia of mice infected with *C. albicans*, *S. pneumoniae* and H1N1. Score points were recorded on spreadsheets.

### Time of event

The log-rank test was used to analyze mouse survival^37^. The time of event is the time of death with or without euthanasia (Supplementary Tables 1 and 2), measured in days post-infection. To explore the impact of the threshold at which weight loss leads to euthanasia, a new time of event was computed for each mouse regarding its maximum weight loss during the experiment. A time of event was calculated for thresholds varying from 5 to 30% weight loss with an increment of 1%. The computation was performed in python 3.10.6 and data are available on GitHub (https://github.com/mbrochut/weight_loss).

### Statistical analysis

To evaluate the relationship between weight loss and health status, we used data from cohort 2, where both parameters were available. The maximum weight loss was plotted against the highest clinical score. The Pearson correlation coefficient and associated *p* values were calculated using pearsonr from the SciPy v1.12.0 library. To test the distribution of mice that did (*survivors*) and did not (*non-survivors*) survive infection for different thresholds, we used the Pearson’s chi-squared test (${\chi }^{2}$). A test of ${\chi }^{2}$ was applied for each model of infection, and for each threshold of interest: 10%, 15%, 20% and 25%. To analyze the difference in weight loss over time between mice that survived or not during the experiment, a Kruskal–Wallis test was used day-by-day and a mixed effects model was computed using statsmodels 0.14.0 Python package to evaluate the effect of weight loss on multiple days simultaneously. Due to the nature of the computation of mixed effect model that excludes data with premature death, data from the first 4–8 days of the experiments were fitted to include a maximum of mice and to avoid a survival bias. A complete model using data collected all days of the experiments was also fitted. *p* values < 0.05 were considered to be statistically significant.

### Power analysis

Euthanasia based on weight loss influences statistical results. First, a mouse can end up in the dead or alive groups if we modify the threshold leading to sacrifice. Second, the survival time alters the results of the log-rank test. We defined the rejection of the null hypothesis (${H}_{0}$, hypothesis of no difference between groups) in favor of the alternative hypothesis (${H}_{1}$, hypothesis of an effect size between groups) when the *p* value of a test is lower than 0.05^38^. The rejection of the null hypothesis is directly related to the power (*P*), which is the probability to reject ${H}_{0}$ in favor of ${H}_{1}$ knowing there is a difference between groups, i.e. ${H}_{1}$ is true^39^:

1 $$Power=P\left({H}_{0}|{H}_{1}\right)$$

We can express this probability in terms of a fraction, defined by the number of times we have rejected ${H}_{0}$ in favor of ${H}_{1}$ divided by the total number of *p* values (#*p*) that were calculated:

2 $$Power=P\left({H}_{0}|{H}_{1}\right)=\frac{( \#p <0.05)}{\#p}$$

Power calculation is meaningful when there is an expected effect size to be tested between groups. Power was investigated using in-house data-based calculation or simulation-based calculation. The in-house data-based calculation used data from experiments carried out in the laboratory with a control and a treatment group of mice, excluding experiments to determine the LD_50_ (228 mice) and experiments for which no differences between groups were expected (271 mice)^40^. We performed log-rank tests using survival data with sacrifice thresholds varying from 1 to 1%, from 5 to 30%. The number of *p* values < 0.05 was used to run power calculation using Eq. (2). Each experiment was treated independently. The simulation-based calculation was carried out using the data from all the experiments performed within one specific model of infection. Since power is reflected by the number of significant *p* values, we created artificial experiments. First, we set the number (N) of mice used per group and chose a type of infection. Second, we randomly selected N mice from the control and the treatment groups. Finally, we compared the newly generated groups by computing *p* values with the log-rank test. The process was run 5000 times and repeated 20 times for a total of 100,000 tests to derive a theoretical power at N mice. We increased the number of mice in each group and fitted the data with a curve created with scipy 1.9.0 to calculate the number of mice corresponding to a power of 80%. With the number of mice thus obtained, we computed with the same principle 100,000 new *p* values with variable weight loss thresholds, incremented by 1% from 5 to 30% of weight loss.

## Results

### Data collection

To assess the relationship between weight loss and outcome of infected mice, and to define whether thresholds leading to euthanasia can be optimized, we performed a retrospective analysis of data recorded during experiments carried out between June 2014 and March 2023. We focused on models of infection used in more than 5 experiments and with over 200 mice. Among 2400 mice, 791 and 1609 mice were used under authorizations covering the years 2014–2018 (cohort 1) and 2018–2023 (cohort 2), respectively. We excluded 43 mice from the analyses because they died after anesthesia (39) or were incorrectly injected with the infectious agent (4) (Fig. 1). The models of infection included (number of experiments, number of mice) systemic infection with *Candida albicans* (6, 252) and *Listeria monocytogenes* (39, 1048), and intranasal infection with *Streptococcus pneumoniae* (32, 721) and H1N1 (19, 336) (Table 1). The infectious doses (median, interquartile range [IQR]) were 1.6 × 10^5^ [0.8–2.0 × 10^5^] colony forming units (CFU) *C. albicans*, 1.2 × 10^5^ [0.9–1.6 × 10^5^] CFU *L. monocytogenes*, 5.8 × 10^4^ [0.2–2.9 × 10^5^] CFU *S. pneumoniae* and 4.0 × 10^4^ [0.2–4.0 × 10^4^] plaque forming units H1N1. We compared groups of control mice (wild-type mice) and experimental mice (with immunomodulatory treatment in wild-type mice, or knockout mice). The mortality rate in the control groups was 40.2% for *C. albicans*, 67.9% for *L. monocytogenes*, 71.5% for *S. pneumoniae* and 51.5% for H1N1 infection. Over the period of data acquisition, the inoculum could vary depending on our working hypothesis. When our objective was to test an intervention likely to increase mortality, we chose a sublethal inoculum in control mice. Conversely, when our objective was to test an intervention likely to reduce mortality, we chose an inoculum that would induce high mortality in the control group.

**Table 1:** Characteristics of the experimental models of infection and associated mortality and weight loss.

Infectious agent	Experiments (n)	Mice (n)	Inoculum, median CFU/PFU [IQR]	Non-survivors (n)	Mortality rate (%)	Median time to death (days)	Median time to death of non-survivors (days)	Mice with > 30% weight loss (n)	Survivors with > 30% weight loss (n)
*C. albicans*	6	252	1.6 × 10^5^ [0.8–2 × 10^5^]	88	34.9	n.a	8	64	16
*L. monocytogenes*	39	1′048	1.2 × 10^5^ [0.9–1.6 × 10^5^]	493	47.0	n.a	4	8	5
*S. pneumoniae*	32	721	5.8 × 10^4^ [0.2–2.9 × 10^5^]	393	54.5	9	5	2	1
H1N1	19	336	4.0 × 10^4^ [0.2–4.0 × 10^4^]	133	39.6	n.a	7	68	17
Total	96	2'357		1'107				142	39

*CFU* colony forming unit, *IQR* interquartile range, *n.a.* not applicable, *PFU* plaque forming unit.

### Weight loss to predict outcome

**Fig. 2:**
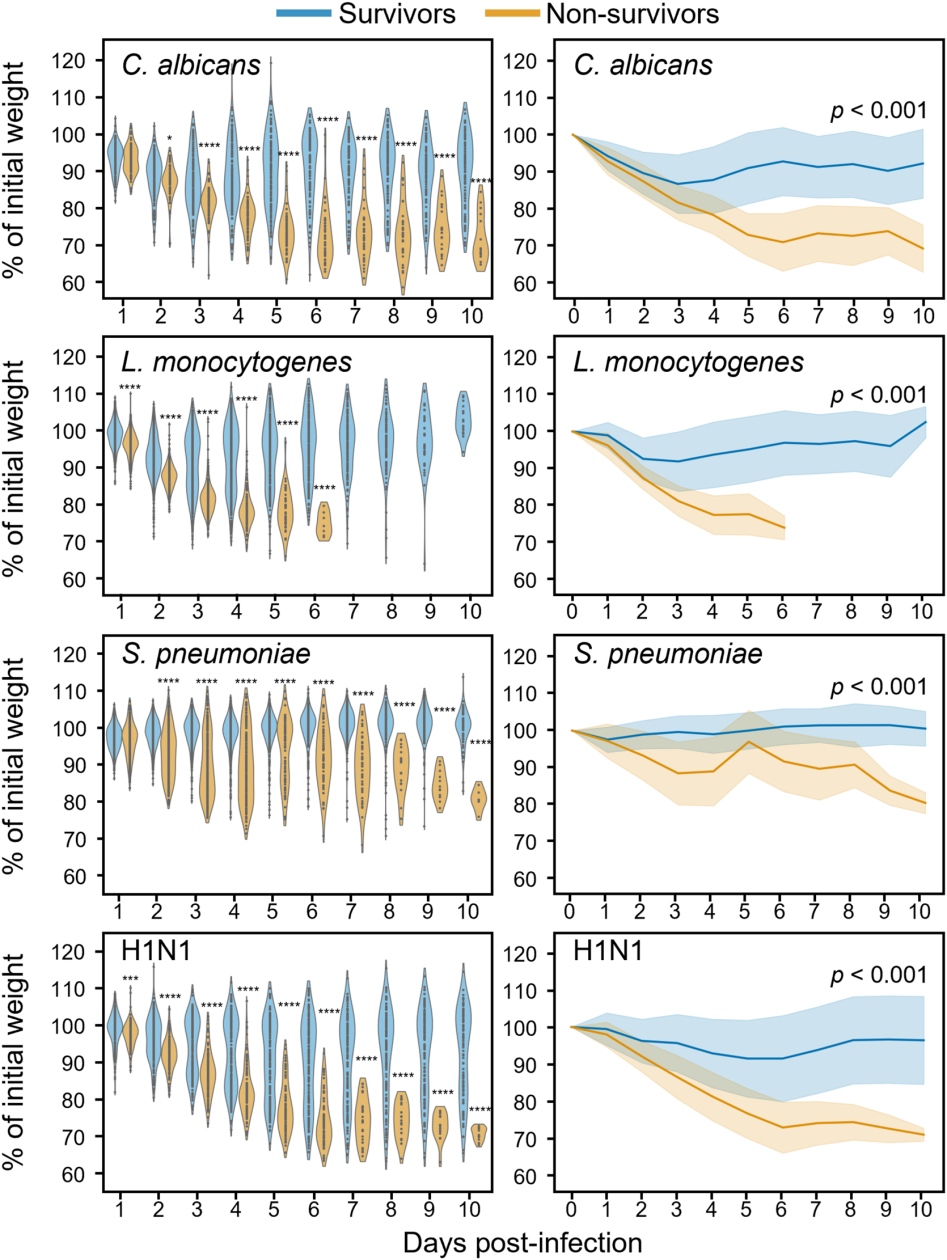
Percent of initial weight of mice infected with *C. albicans*, *L. monocytogenes*, *S. pneumoniae* and H1N1. (**A**) Day-by-day data were compared with the Kruskal–Wallis test. Each dot represents one mouse. *p < 0.05, ***p < 0.001; ****p < 0.0001 (survivors *versus* non-survivors at one specific day). (**B**) Median of percent weight loss with standard deviation as bandwidth. Blue: mice that survived; orange: mice that did not survive. Statistical significance of weight loss over time was computed by mixed effect model (*p* < 0.001).

Taking all the mice together, the proportion of mice that did not survive infection, either because they died or were euthanized, was higher for *L. monocytogenes* (47.0%) and *S. pneumoniae* (54.5%) than for *C. albicans* (34.9%) and H1N1 (39.6%) (Table 1 and Supplementary Fig. 1). The median survival time was 9 days for *S. pneumoniae* infection. In all models, independently of the conditions applied to the mice (control versus experimental group), weight loss segregated mice that survived from mice that did not survive. Differences were statistically significant as early as one day post-infection with *L. monocytogenes* and H1N1, and two days post-infection with *C. albicans* and *S. pneumoniae* (Fig. 2, left). Accordingly, the mean body weight loss over time showed a clear differentiation between surviving and non-surviving mice (Fig. 2, right). The percentage of initial weight (mean ± standard deviation) at day 6 post-infection reached 74.6 ± 3.2% (n = 7), 74.4 ± 6.9% (n = 66), 72.8 ± 7.9% (n = 51), and 91.9 ± 8.1% (n = 57) in mice dying from *L. monocytogenes*, H1N1, *C. albicans* and *S. pneumoniae*, respectively.

To further compare weight loss and survival rate after infection, we performed a mixed-effects analysis to examine the relationship between weight loss and survival rate across multiple time points while considering individual differences among mice. The model was computed from day 0 to day 3 (*S. pneumoniae* and *L. monocytogenes*) and to day 5 (*C. albicans* and H1N1) to include as many data points as possible while minimizing bias due to missing values. Highly statistically significant differences (*p* < 0.001) were observed between groups of survivors and non-survivors (Fig. 2). Differences were also statistically significant when the mixed-effects analysis was performed using all data points (*p* < 0.001, data not shown).

Since data were collected from cohorts running from 2014 to 2018 without weight loss limit (cohort 1) and from 2018 to 2023 with specified weight loss leading to the euthanasia (cohort 2) (Supplementary Tables 1 and 2), we wondered whether we introduced a bias towards representing more non-survivors using the second cohort. To clarify this aspect, we compared the mortality rates of the first and second cohorts. The mortality rate was similar and not statistically significant for each of the models of infection (Supplementary Fig. 2). Moreover, the proportion of mice that reached more than 30% weight loss was similar in the cohorts 1 and 2 (Supplementary Table 3).

### Importance of weight loss in clinical score to predict outcome

**Fig. 3:**
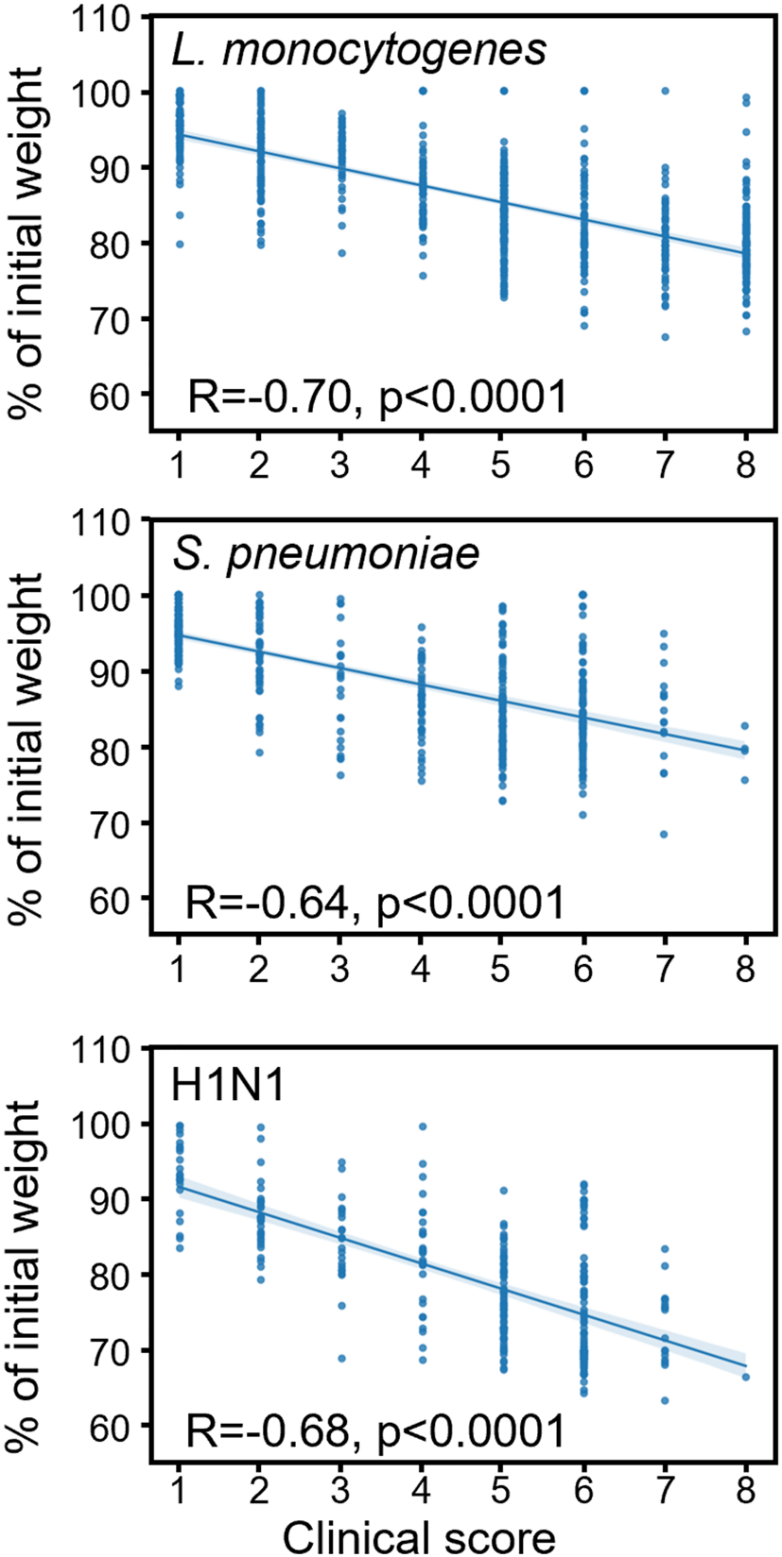
Correlation between weight loss and clinical scores. The maximum weight loss was plotted against the highest clinical score for non-survivors from cohort 2. The Pearson correlation coefficient (R) and *p* values were calculated using pearsonr.

Weight loss (ranked 0–3) is one among other criteria to calculate the clinical score. Indeed, septic mice show external signs of illness affecting their mobility and aspect (behavior, ruffled coat, diarrhea, conjunctivitis) that are recorded on score sheets (Supplementary Tables 1 and 2). So, we could analyze the relationship between clinical scores and weight loss using the data collected in cohort 2 (i.e. without *C. albicans*) where both parameters were available. As shown in Fig. 3, the clinical score and the percentage of initial weight (reflecting weight loss) inversely correlated for *L. monocytogenes* (R = − 0.70, *p* < 0.0001), *S. pneumoniae* (R = − 0.64, *p* < 0.0001) and H1N1 (R = − 0.68, *p* < 0.0001) infections.

We then further explored the relative contribution of weight loss and clinical score (which includes weight loss) in promoting euthanasia. Table 2 summarizes the data collected from euthanized mice in cohort 2. The proportion of mice reaching the weight loss limit for euthanasia (i.e. 3) while having a clinical score not requiring euthanasia (i.e. < 6) was 13.9%, 0.6% and 11.7% for *L. monocytogenes*, *S. pneumoniae* and H1N1 infection, respectively. The proportion of mice achieving a clinical score requiring euthanasia (i.e. ≥ 6) was much higher, at 86.1%, 99.4% and 88.3% for *L. monocytogenes*, *S. pneumoniae* and H1N1, respectively. However, within these groups, it is interesting to note that 54–56% of mice infected by *L. monocytogenes* and H1N1 also reached the maximum weight loss score of 3 imposing euthanasia. In addition, 43.0%, 95.3% and 42.6% of mice with a clinical score ≥ 6 reached a weight loss score of 2 when infected by *L. monocytogenes*, *S. pneumoniae* and H1N1.

**Table 2:** Reasons leading to the euthanasia.

Infectious agent (n)	Reason of euthanasia					
	Weight score = 3, n (%)*	Clinical score ≥ 6, n (%)*				
			Weight score (0–3) used to calculate clinical score, n (%)			
			0	1	2	3
*L. monocytogenes* (216)	30 (13.9)	186 (86.1)	0 (0.0)	5 (2.7)	80 (43.0)	101 (54.3)
*S. pneumoniae* (107)	1 (0.6)	106 (99.4)	1 (0.9)	3 (2.8)	101 (95.3)	1 (0.9)
H1N1 (77)	9 (11.7)	68 (88.3)	0 (0.0)	1 (1.5)	29 (42.6)	38 (55.9)

Data were collected from euthanized mice in cohort 2. Mice that died between two monitoring (mainly during the night) were not considered.

*Weight score = 3: the weight loss limit for euthanasia (3) was reached while the total clinical score was < 6. Clinical score ≥ 6: the clinical score limit for euthanasia (6) was reached while the weight loss score was ≤ 3.

Overall, these results indicate that most of the mice infected with *L. monocytogenes* and H1N1 reached the maximum weight loss threshold earlier or at the same time as the clinical score leading to euthanasia, while the clinical score appears to be reached just before maximum weight loss for *S. pneumoniae*-infected mice.

### Impact of weight loss threshold on the mortality rate

Humane endpoints based on statistical and ethical considerations should quickly relieve the suffering of mice, without euthanizing animals that would otherwise have survived. Hence, we tested whether weight loss thresholds set at 25%, 20%, 15% and 10% increased the mortality rate in the four models of infection.

As expected, the death rate increased as weight loss thresholds were lowered, but from different thresholds onwards (Table 2). In the models of *C. albicans* and H1N1 infection, using a threshold of 25% increased the number of mice euthanized by 33 and 45 (over 252 and 336 mice), and the death rate from 34.9 to 48.0% and from 39.6 to 53.0% (*p* < 0.001), respectively. In the case of *L. monocytogenes* infection, statistical differences were observed from a threshold of 20%, which increased the number of mice euthanized by 79 (over 1048) and the death rate from 47.0 to 54.6% (*p* < 0.001). Finally, in the model of *S. pneumoniae* infection, thresholds of 25–15% had no influence on death rate, whereas a threshold of 10% increased the number of euthanized mice by 53 (over 721) and the death rate from 54.5 to 61.9% (*p* < 0.001). Therefore, the weight loss threshold influences the number of mice euthanized depending on the model of infection (Table 3).

**Table 3:** Calculation of the impact of weight loss threshold on the mortality rate linked to an increase in euthanasia.

Model of infection (total mice used, observed mortality in %)	Minimum weight loss (%) leading to euthanasia^1^											
	25%			20%			15%			10%		
	Additional deaths (n)	Death rate (%)	*p* value*	Additional deaths (n)	Death rate (%)	*p* value	Additional deaths (n)	Death rate (%)	*p* value	Additional deaths (n)	Death rate (%)	*p* value
*C. albicans* (252, 34.9%)	33	48.0	< 0.001	61	59.1	< 0.001	99	74.2	< 0.001	132	87.3	< 0.001
*L. monocytogenes* (1048, 47.0%)	25	49.4	0.122	79	54.6	< 0.001	184	64.6	< 0.001	283	74.0	< 0.001
*S. pneumoniae* (721, 54.5%)	4	55.1	0.764	10	55.9	0.453	22	57.6	0.097	53	61.9	< 0.001
H1N1 influenza virus (336, 39.6%)	45	53.0	< 0.001	76	62.2	< 0.001	116	74.1	< 0.001	150	84.2	< 0.001

^1^For each new weight loss threshold, the number of additional deaths resulting from euthanasia and the resulting death rate have been calculated.

*p values comparing observed and estimated mortality rates were calculated using the Chi-squared test.

### In-house data-based power calculation

**Fig. 4:**
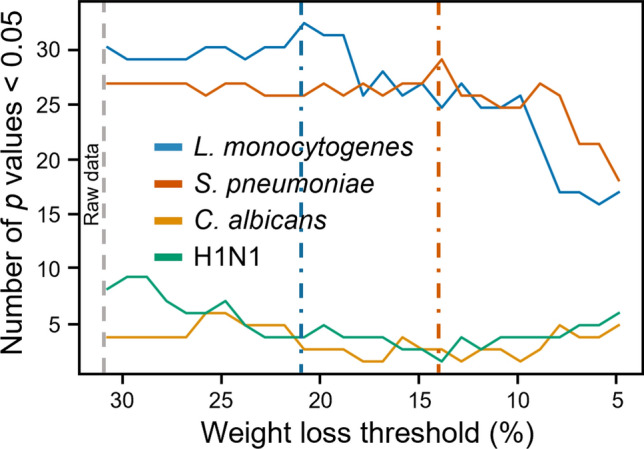
Power analysis using the *in-house* approach. Power calculation was performed post hoc using the *in-house* approach (see “Methods”, “Power analysis”). Sacrifice thresholds were set from 1 to 1% to perform log-rank tests, count the number of *p* < 0.05, and run power calculation using Eq. (2). The orange and blue dotted lines mark the weight loss threshold after which the power decreases for *L. monocytogenes* and *S. pneumoniae* infections.

Power calculations are conducted to estimate the size of the samples to be used in the experiments. Numerous experimental conditions, including the strain, dose, and route of inoculation of the microorganism, as well as the treatment, genetic background, sex, and age of the mice influence computing power. As a first step, we assessed the power post hoc, based on the number of *p* < 0.05 reported to the total number of tests performed using data obtained in experiments comparing a control and a treatment group of mice (see “Methods”, “Power analysis”). Each model of infection was evaluated separately, using new survival data obtained with sacrifice thresholds varying from 1 to 1% (Fig. 4). We observed a decline in power from a weight loss of 21% in experiments using *L. monocytogenes*. In the model of *S. pneumoniae* infection, power was rather unstable between 22 and 14%. The number of *p* < 0.05 decreased rapidly after 14%. As anticipated, due to the limited number of experimental data, we did not observe noticeable changes in power in the models of infection with *C. albicans* and H1N1. More data should be collected to investigate power in these models.

### Simulation-based power calculation

**Fig. 5:**
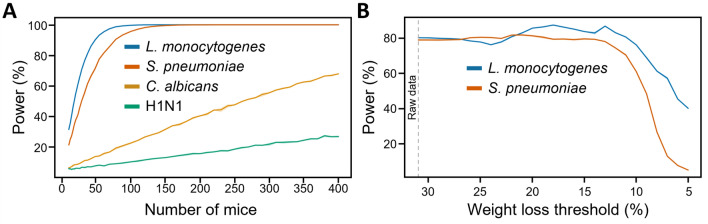
Simulation-based power calculation. (**A**) Simulation to determine the number of mice needed to reach a power of 80%. (**B**) Simulation of power with 35 (*L. monocytogenes*) and 56 (*S. pneumoniae*) mice as a function of weight loss.

Simulation is a powerful tool to increase the number of datapoints without the requirement of additional experimental mice. Hence, we computed experiments involving control and treated mice randomly selected from our dataset. First, we determined the number of mice, between 10 and 400, needed to achieve a power of 80% for a given type of infection (Fig. 5A). Simulation resulted in curves that reached a power of 80% with 35 and 56 mice in models of infection with *L. monocytogenes* and *S. pneumoniae*. Simulations with *C. albicans* and H1N1 were not conclusive due to an insufficient effect size to determine a power of 80% (Fig. 5A). We then selected 35 (*L. monocytogenes*) and 56 (*S. pneumoniae*) mice per group to run simulations varying the threshold of sacrifice (Fig. 5B). For *L. monocytogenes*, the power was stable between 30 and 20% weight loss and increased slightly to 87.3% until a weight loss of 18%. Power decreased rapidly to 40.0% from 13 to 5% weight loss. For *S. pneumoniae*, the power remained stable up to 13% weight loss and decreased rapidly thereafter, reaching 5.2% at 5% weight loss. Overall, in-house data-based and simulation-based power calculations revealed variability and/or reduction of power as weight loss thresholds approached 20% for *S. pneumoniae* and *L. monocytogenes* models.

## Discussion

The implementation of appropriate humane endpoints is an ethical foundation for experimental research using animals. We report here the most extensive study, to our knowledge, exploring the relationship between weight loss threshold and outcome of mice used in preclinical models of sepsis. Globally, our data indicate that weight loss is a valuable predictor of mortality, but that specific thresholds should be defined for each model of infection. Hence, weight loss is an objective, easy and powerful measure for generating composite clinical scores to minimize mouse suffering while preserving the statistical power of the experiments.

Our study is unique because it is based on an important data set collected in experiments testing working hypothesis linked to host response to infections. In this sense, it differs from tightly controlled studies specifically designed to test the predictive value of weight loss (with or without other criteria of wellbeing) during infections^7–9,11,41–44^. We analyzed data accumulated over 10 years, excluding those from experiments with a low number of data points, i.e. one experiment with *Citrobacter rodentium* (n = 15 mice), one with *Staphylococcus aureus* (n = 10), two with *Pseudomonas aeruginosa* (n = 68), seven with *Escherichia coli* (n = 116) and two with lipopolysaccharide (LPS, n = 37). Even for a specific model of infection, several factors varied including inoculum size, mouse characteristics (strain, sex, age, origin, microbiota, treatment), housing conditions (temperature, humidity, number of mice per cage, enrichment material), as well as animal keepers and experimenters and the criteria used to evaluate animal welfare. On the one hand, these factors may be confounding and influence our analyses. On the other hand, they reflect the diversity of biomedical research and, in our opinion, make this study all the more interesting.

A small number of mice achieved 30% weight loss while surviving (39 out of 2357 mice, 1.7%, Table 1), suggesting that an important weight loss is predictive of poor outcome^11^. Though, weight loss does not always predict death. Among the 4 models of infections, infections with *C. albicans* or H1N1 resulted in the greatest weight loss in both survivor and non-survivor groups, while they were the least lethal (35–40% mortality with *C. albicans* and H1N1 *versus* 47–54% mortality with *L. monocytogenes* and *S. pneumoniae*). This is because acute infections result in more rapid mortality and leave less time for substantial weight loss compared with subacute or chronic infections. Accordingly, mice that did not survive *S. pneumoniae* infection lost on average 15% of body weight, while those that survived lost 6%. Importantly, for a specific model of infection and according to our working hypothesis, we used an inoculum with mortality rates ranging from 0 to 100% in the control group. Hence, inoculum also has an impact on the timing and trajectory of weight loss.

Considering non-surviving mice in each of the four models of infection, the mean time to death was 8 and 7 days for *C. albicans* and H1N1 infections, while it was only 4 and 5 days for *L. monocytogenes* and *S. pneumoniae* infection (Table 1 and Supplementary Fig. 1). Sixteen of 64 (25.0%) and 17 of 68 (25.0%) mice infected with *C. albicans* and H1N1 that lost more than 30% of their initial weight at some point during the experiment finally survived. Fixing a maximum weight loss of 20% would have increase the proportion of non-survivors due to increased euthanasia by 24% for *C. albicans* and 22% for H1N1 infections.

Given our large dataset, we could analyze the effect size by performing post hoc in-house data-based power calculation. Unfortunately, for *C. albicans* and H1N1 infection, the number of *p* values calculated was not sufficient to draw a conclusion. For *L. monocytogenes* and *S. pneumoniae*, we observed a decrease in power at around 20% weight loss, but also variability in power. This is not surprising since calculations were based on log-rank tests with small samples (n = 7/group on average). To solve this issue, we employed simulation-based power calculation carried out with 100,000 tests instead of around 40 tests for in-house data-based power calculation, which greatly reduced power variability. In simulation-based power calculation, power decreased sharply at around 13% weight loss in models of infection with *S. pneumoniae* and *L. monocytogenes*. Consequently, the introduction of stringent thresholds would have important consequences on designing the size of the experimental groups.

Weight loss was not reflective of mortality in a study assessing novel methods to evaluate the outcome of mice subjected to intranasal infection with *Enterococcus faecium, Staphylococcus aureus, Klebsiella pneumoniae, Acinetobacter baumannii, Pseudomonas aeruginosa, Enterobacter cloacae* and *Escherichia coli*^11^. Instead, the measurement of internal and external temperature using microchips and infrared thermometer revealed that internal temperature more precisely predicted mortality. Taken together with our data, these observations comfort the idea that weight loss recommendations for euthanasia cannot be universal and that a threshold of 20%, as is often used, is not applicable to any type of infection^13^. For instance, we now use a weight loss of ≥ 20%, instead of ≥ 30%, as a humane endpoint for *S. pneumoniae* infected mice. On the contrary, we increased the weight loss limit used to euthanize *L. monocytogenes*-infected mice from ≥ 20 to ≥ 25%.

Our composite clinical scores include weight loss in addition to mobility and external aspect (supplementary Tables 1 and 2). If we focus on euthanized animals from cohort 2, 68.2% of *Listeria* and 67.6% of H1N1-infected mice were euthanized reaching the weight loss humane endpoint (Table 2). On the contrary, 98.5% of *S. pneumoniae*-infected mice were euthanized because of their clinical score, while weight loss was almost invariably scored at 2. These observations support further our decision to reduce the weight loss limit leading to euthanasia of *S. pneumoniae*-infected mice. All these considerations imply that changes in the weight loss threshold are not trivial, and that the question of whether the clinical score requires euthanasia before a fixed weight loss limit must be assessed on a case-by-case basis according to the score sheet criteria (number of dimensions, scoring system, weight of each criterion). If the weight loss thresholds for euthanasia are very strict, then weight loss would be the main criterion for stopping the experiment. On the contrary, if the weight loss thresholds are permissive, the weight loss limit will probably never be reached in sacrificed mice. Based on our analyses, we recommend that thresholds should be defined in a model-dependent manner. Though, we recognize that it is challenging to collect a large amount of data points to perform statistical and power calculations. While using weight loss as a humane endpoint is debated^11,12^, weight loss is a valuable indicator of the health status of mice when combined with other criteria such as temperature, appearance, and behavior.

Several studies reported humane endpoints in preclinical mouse models of infection and sepsis. Body temperature was a predictor of outcome of bacterial and fungal infections, as well as cecal ligation and puncture-induced sepsis^11,42,45–47^. However, there are important variations in temperature cut-off values between studies^10^. A sepsis severity score based on grading appearance (smooth or ruffled fur), eye aspect (normal, with secretion), activity (active, lethargic, moribund), consciousness (alert, sleepy) and respiration (normal or slow breaths) had a good predictive value in mice infected intratracheally with *K. pneumoniae*^48^. A similar score based on subjective parameters (consciousness, activity, behavior, response to stimuli and breathing) was improved by adding objectives values (glycemia, temperature, body weight). The combinatorial score achieved high sensitivity and specificity for the early detection of sepsis in mice injected intraperitoneally with fecal solution^43^. In a machine learning approach using data from mice challenged with LPS, scores of sickness and temperature improved the accuracy and performance of the prediction model^10^. Given that the host response to live microorganisms differs from that to toxins such as LPS, a similar learning approach using data from infected mice could prove instructive. Generally, the scores reported in the literature have been established as part of specific studies. It is assumed that humane endpoints may vary according to the infectious model, disease intensity, the treatments administrated to the mice, and many additional factors including age, sex, and environmental conditions. Therefore, the robustness of the scores must be verified in independent experimental contexts so that they can be used as humane endpoint for rapidly terminating sepsis experiments while ensuring that the scientific objectives are achieved.

Our study has several limitations. We excluded from our analysis experiments with few data points, which could have been informative. We did not have groups of uninfected mice for each model to adhere to the 3Rs, but that raises the question of the influence of anesthesia and mouse handling on weight loss. In response, mice anesthetized with ketamine and xylazine and receiving a non-lethal inoculum of H1N1 did not lose weight (100.2% ± 2.7% of initial weight 24 h after treatment). Moreover, in all experiments, the control and treated groups were manipulated in the same way (injection of diluent for the control group). These observations suggest that, apart from deleterious infections, handling procedures had a relatively minor impact on weight loss. We included data collected during experiments carried out with different parameters, including the parameters used to monitor the wellbeing of the mice and to terminate the experiments. It is possible that we have overestimated the weight limits associated with mortality. Nonetheless, we believe that the diversity of our data reflects the situation encountered in many laboratories and is closer to reality than the studies specifically designed to test the predictive value of a predefined humane endpoint.

To conclude, we report the largest study analyzing weight loss in models of bacterial, viral, and fungal infections in real world settings. Our data support the idea that weight loss thresholds used as humane endpoint should be adapted to the experimental model used. Whereas preclinical mouse models remain the best way to decipher the complex biological processes underlying sepsis and to test new treatment options, they must be conducted with strict ethical considerations in mind. From this point of view, our calculations allowed us to adjust the weight loss thresholds to minimize mice suffering without increasing unreasonably the size of the experimental groups. Thus, we recommend an infection-model-dependent evaluation of weight loss thresholds used in preclinical models of sepsis.

## Supplementary Information

Supplementary Information.

## Data Availability

All data generated or analyzed during this study are included in this article and its supplementary information files or have been deposited at https://github.com/mbrochut/weight_loss.

## Abbreviations

CFU: Colony forming unit
IV: Influenza virus
LD_50_: Lethal dose 50
LPS: Lipopolysaccharide
