# Author Correction: Using weight loss to predict outcome and define a humane endpoint in preclinical sepsis studies

**DOI:** 10.1038/s41598-024-76906-9

**Published:** 2024-10-24

**Authors:** Maëlick Brochut, Tytti Heinonen, Tiia Snäkä, Charly Gilbert, Didier Le Roy, Thierry Roger

**Affiliations:** Infectious Diseases Service, Department of Medicine, Lausanne University Hospital and University of Lausanne, CLED.04.407, Chemin des Boveresses 155, 1066 Epalinges, Switzerland

Correction to: *Scientific Reports* 10.1038/s41598-024-72039-1, published online 10 September 2024

The original version of this Article contained an error in the Funding section.

“TR was supported by Grants from the Swiss National Science Foundation (SNSF, Grant number 310030_207418), the Fondation Carigest/Promex Stiftung für die Forschung (Geneva, Switzerland), the Horizon 2020 Grant ImmunoSep (number 847422) and HDM-FUN (number 847507).”

now reads:

“TR was supported by Grants from the Swiss National Science Foundation (SNSF, Grant number 310030_207418), Promex Stiftung für die Forschung (Geneva, Switzerland), the Horizon 2020 Grant ImmunoSep (number 847422) and HDM-FUN (number 847507).”

The original Article has been corrected.

