# Supplementary material for: Using weight loss to predict outcome and define a humane endpoint in preclinical sepsis studies
Source: Sci Rep. 2024 Sep 10;14:21150. doi: 10.1038/s41598-024-72039-1 (PMC11387420; doi:10.1038/s41598-024-72039-1)
Supplement: Supplementary file 1 — Supplementary Information. [file 41598_2024_72039_MOESM1_ESM.pdf]

Supplementary information

Using weight loss to predict outcome and define a humane endpoint in preclinical sepsis studies

Maëlick Brochut, Tytti Heinonen, Tiia Snäkä, Charly Gilbert, Didier Le Roy, Thierry Roger

Infectious Diseases Service, Department of Medicine, Lausanne University Hospital and University of Lausanne, Epalinges, Switzerland

Supplementary Tables

Supplementary Table 1. Score sheets used in 2014-2018 (cohort 1).

Criteria	Score	Description
**Mobility + aspect**	0	Normal
	1	Bristly hair
	2	Bristly hair and (conjunctivitis or diarrhea or mobility impairment)
	3	Bristly hair and (conjunctivitis or diarrhea) and mobility impairment
	4	Moribund
**Action**	4	Euthanasia

Scores are calculated depending on observable traits and mobility. Mice are monitored every 8 to 24 hours. Weight is measured at the same time as the score is evaluated.

Supplementary Table 2. Score sheets used in 2018-2023 (cohort 2).

Criteria	Score	Description
**Mobility**	0	Normal
	1	Slower movement
	2	Hunched posture
	3	Moribund or uncontrolled movement
**Aspect**	0	Normal
	1	Bristly hair or conjunctivitis or diarrhea
	2	Bristly hair and conjunctivitis or diarrhea
**Weight**	0	No loss
	1	< 10% loss
	2	≥ 10% & <20% (*L. monocytogenes*) or <30% (other agents) loss
	3	Weight loss ≥ 20% (*L. monocytogenes*) or ≥ 30% (other agents)
**Action**	0-1	Monitored 3 times a week
	2-3	Monitor once a day
	4-5	Monitor twice a day
	Total of 6, or 3 in mobility or 3 in weight	Euthanasia

Scores are calculated depending on mobility, observable traits (bristly hair, conjunctivitis, diarrhea) and weight loss. The action column (calculated automatically in excel spreadsheets while recording sub-scores) indicates the actions to be taken according to the cumulative score of the criteria mobility, aspect, and weight.

Supplementary Table 3. Number and proportion of mice achieving > 30% weight loss in cohorts 1 and 2.

	Cohort 1 (2014-2018)			Cohort 2 (2018-2023)		
	Total mice (n)	Mice with > 30% weight loss (n)	Mice with > 30% weight loss (%)	Total mice (n)	Mice with > 30% weight loss (n)	Mice with > 30% weight loss (%)
L. monocytogenes	263	4	1.5	785	4	0.5
S. pneumoniae	211	1	0.5	510	1	0.2
H1N1	63	12	19.0	273	56	20.5

Supplementary Figures

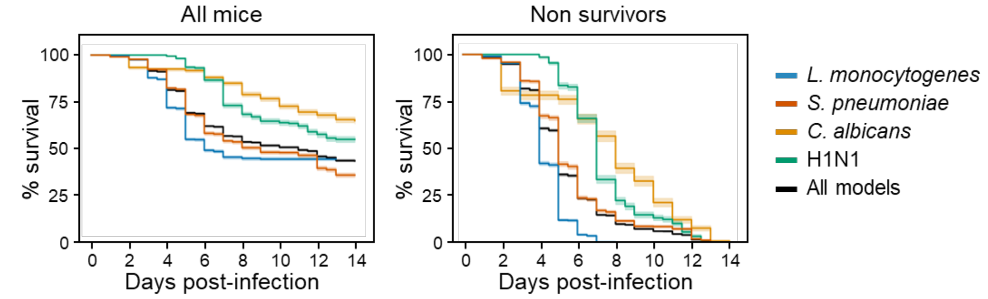

Supplementary Figure 1. Survival of mice used in preclinical models of sepsis. Kaplan-Meier curves of mice (total/non-survivors) infected with *L. monocytogenes* (1'048/493), *S. pneumoniae* (721/393), *C. albicans* (252/88) and H1N1 (336/133). Black curves represent data obtained with mice from all models.

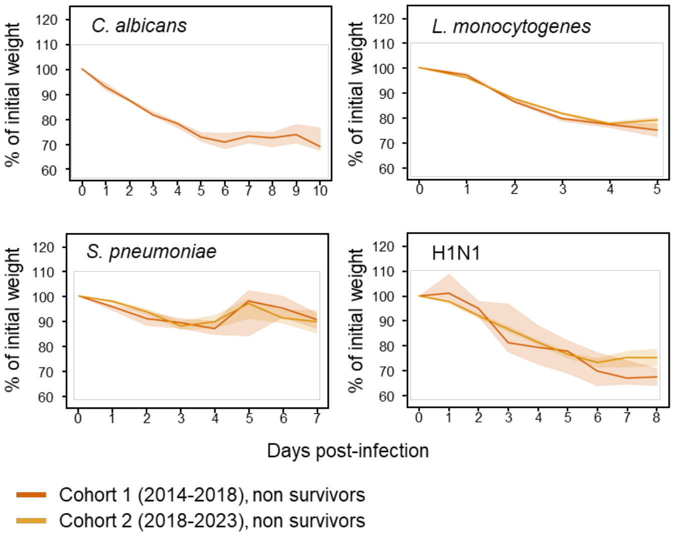

Supplementary Figure 2. Percent of initial weight of mice dying from infection by *C. albicans*, *L. monocytogenes*, *S. pneumoniae* and H1N1. Data from cohort 1 (2014-2018, red) and cohort 2 (2018-2023, orange) are expressed as median % weight loss with standard deviation as bandwidth. *C. albicans* infections were present in the cohort 1 only. The statistical difference between the curves after computing a mixed-effect model was $p > 0.05$.
